# Sleep quality and temperament in association with autism spectrum disorder among infants in Japan

**DOI:** 10.1038/s43856-023-00314-9

**Published:** 2023-06-16

**Authors:** Kimiyo Kikuchi, Takehiro Michikawa, Seiichi Morokuma, Norio Hamada, Yoshiko Suetsugu, Subaru Ikeda, Kazushige Nakahara, Kiyoko Kato, Masayuki Ochiai, Eiji Shibata, Mayumi Tsuji, Masayuki Shimono, Toshihiro Kawamoto, Shouichi Ohga, Koichi Kusuhara, The Japan Environment and Children’s Study Group, Michihiro Kamijima, Shin Yamazaki, Yukihiro Ohya, Reiko Kishi, Nobuo Yaegashi, Koichi Hashimoto, Chisato Mori, Shuichi Ito, Zentaro Yamagata, Hidekuni Inadera, Takeo Nakayama, Hiroyasu Iso, Masayuki Shima, Youichi Kurozawa, Narufumi Suganuma, Takahiko Katoh

**Affiliations:** 1 Department of Health Sciences, Graduate School of Medical Sciences, Kyushu University, Fukuoka, Japan; 2 Department of Environmental and Occupational Health, School of Medicine, Toho University, Tokyo, Japan; 3 Research Center for Environment and Developmental Medical Sciences, Kyushu University, Fukuoka, Japan; 4 Department of Obstetrics and Gynecology, Graduate School of Medical Sciences, Kyushu University, Fukuoka, Japan; 5 Department of Pediatrics, Graduate School of Medical Sciences, Kyushu University, Fukuoka, Japan; 6 Regional Center for Japan Environment and Children’s Study, University of Occupational and Environmental Health, Kitakyushu, Japan; 7 Department of Obstetrics and Gynecology, School of Medicine, University of Occupational and Environmental Health, Kitakyushu, Fukuoka Japan; 8 Department of Environmental Health, School of Medicine, University of Occupational and Environmental Health, Kitakyushu, Fukuoka Japan; 9 Department of Pediatrics, School of Medicine, University of Occupational and Environmental Health, Kitakyushu, Fukuoka Japan; 10 Nagoya City University, Nagoya, Japan; 11 National Institute for Environmental Studies, Tsukuba, Japan; 12 National Center for Child Health and Development, Tokyo, Japan; 13 Hokkaido University, Sapporo, Japan; 14 Tohoku University, Sendai, Japan; 15 Fukushima Medical University, Fukushima, Japan; 16 Chiba University, Chiba, Japan; 17 Yokohama City University, Yokohama, Japan; 18 University of Yamanashi, Chuo, Japan; 19 University of Toyama, Toyama, Japan; 20 Kyoto University, Kyoto, Japan; 21 Osaka University, Suita, Japan; 22 Hyogo College of Medicine, Nishinomiya, Japan; 23 Tottori University, Yonago, Japan; 24 Kochi University, Nankoku, Japan; 25 Kumamoto University, Kumamoto, Japan

**Keywords:** Paediatric research, Autism spectrum disorders

## Abstract

**Background:**

Sleep problems and irritable temperaments are common among infants with autism spectrum disorder (ASD). The prospective association between such sleep problems and irritable temperaments and ASDs needs to be determined for elucidating the mechanism and exploring the future intervention study. Thus, in this study, we investigated whether sleep quality and temperament in 1-month-old infants are associated with the onset of ASD in 3-year-old children. We also assessed its sex-stratified associations.

**Methods:**

We conducted a longitudinal study using data from 69,751 mothers and infants from a large-cohort study, the Japan Environment and Children’s Study. We examined the prospective association between infant sleep quality and temperament at 1 month of age and ASD diagnosis by 3 years of age.

**Results:**

Here we show infants with longer daytime sleep have a higher risk of later ASD than those with shorter daytime sleep (risk ratio [RR]: 1.33, 95% confidence interval [CI]: 1.01–1.75). Infants who experienced intense crying have a higher risk of ASD than those who did not (RR: 1.31, 95% CI: 1.00–1.72). There is a difference in sex in the association between a bad mood and later ASD. In particular, female infants experiencing bad moods have a higher risk of ASD than others (RR: 3.59, 95% CI: 1.91–6.75).

**Conclusions:**

The study findings provide important information for future intervention to reduce the risk of future ASD.

## Plain Language Summary

Sleep problems and irritable temperaments are common among infants with autism. This study looked at the sleep and temperament of nearly 70,000 1-month-old infants in Japan and whether they were subsequently diagnosed with autism spectrum disorder during the first three years of life. Children who had slept for longer during the day and were more prone to frequent, prolonged, or intense crying were more likely to have been diagnosed with Autism Spectrum Disorder by age 3. The findings of this study might be useful for those monitoring the development of autism spectrum disorder or developing support for those with autism spectrum disorders.

## Abstract (web summary)

Kikuchi, Michikawa et al. investigate whether sleep quality and temperament in 1-month-old infants is associated with Autism Spectrum Disorder (ASD) in 3-year-old children in Japan. Infants who had longer daytime sleeps and more intense crying had a higher risk of ASD at age 3.

## Introduction

Autism spectrum disorder (ASD) is a neurodevelopmental condition mainly characterized by social communication deficits as well as restrictive and repetitive sensorimotor behaviors^1^. ASD is the unified diagnostic name in the diagnostic and statistical manual of mental disorders (DSM-5)^2^, the diagnostic manual for mental disorders. The prevalence of ASD is 1.09–436 per 10,000 persons (median, 100/10,000 persons) according to a systematic review conducted in 34 countries, which included children- and adult-targeted studies^3^. ASD is often diagnosed as early as 18–24 months of age and coexists with other developmental disorders, such as attention-deficit/hyperactivity disorder and Fragile X, as well as with temperamental characteristics such as anxiety, and irritability^4–6^. The risk factors for ASD include environmental and genetic factors. Known factors include male sex, older siblings’ ASD status, advanced parental age at birth^7,8^, maternal antidepressant medication use^9^, maternal obesity or diabetes^10^, preterm birth, and low birth weight^11^.

Early identification and intervention of signs of autism may reduce future autism symptoms^12^. Adaptive behaviors and expressive and receptive language skills are considered positive consequences of early interventions^13^. Previous studies have identified the early anatomical, functional, and behavioral signs of ASD. The signs of autism identified before 1 year old involve motor and visuoperceptual functions, such as lack of eye contact, pointing, facial expression, and reaction to loud noises^14–17^. Other studies have identified different structures, such as high fractional anisotropy^18,19^ and increased cerebrospinal fluid^20^, involved in brain function before 1 year of age among those who later developed ASD. The study of high risk-siblings later diagnosed with autism showed a decline in play and communication and impaired vocal imitation^21^. The sleep quality and temperament of infants have been suggested as risk factors for ASD. For example, night-awakening at 12 months was associated with autistic traits at 24 months^22^. In the study following children from 1.5 to 9 years old, children with increasing sleep problem trajectory and children with stable and moderate sleep problems had higher levels of autistic traits^23^. Diverse temperament features have also been associated with ASD^24–26^. Infants who were later diagnosed with ASD more frequently exhibited crying patterns different from other developmental delay patterns^27^ or produced pain-related cries with a higher pitch and wider frequency range at 6 months of age^28^. Additionally, the relationship between sleep and ASD at 1 month of age was suggested in a previous study^29^, and its relationship may differ according to infants’ sex as sleep duration and temperaments have been suggested to differ between sexes^30,31^.

To the best of our knowledge, no studies have thoroughly investigated sleep quality and temperament in early infants as predictors of a later ASD diagnosis. Such associations must be ascertained for identifying the mechanism and management of autism. Hence, this study investigates whether the sleep quality and temperament of 1-month-old infants are associated with ASD diagnosis by 3 years old using data from a large-cohort study. We also assess the sex-stratified associations of sleep quality, temperament, and autism. Our findings suggest that sleep quality and temperament are associated with ASD diagnosis.

## Methods

### Study design and participants

**Fig. 1:**
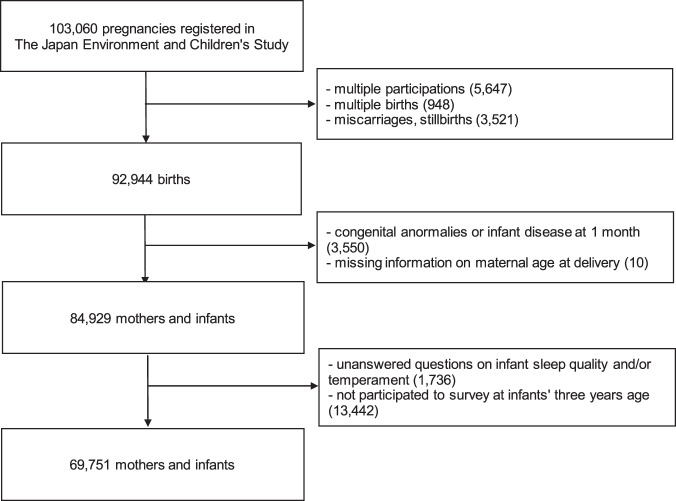
Flow diagram of the selection process of the study participants. Out of 103060 pregnant women registered in the Japan Environment and Children’s Study between January 2011 and March 2014 at Regional Centers, 69751 mothers and infants were selected after excluding those with multiple participations, multiple births, miscarriages or stillbirths, congenital anomalies, missing information on maternal age at delivery, birth at <37 or ≥42 gestational weeks, unanswered questions on infant sleep quality or temperament at 1-month-old, and absence in the survey conducted when the children turned 3 years old.

This longitudinal study investigated the association of infants’ sleep quality and temperament at the age of 1 month with an ASD diagnosis by the age of 3 years. We used data (“jecs-ta-201901930” released in October 2019) from a nationwide prospective birth cohort study, the Japan Environment and Children’s Study (JECS), which is registered in the University Hospital Medical Information Network Clinical Trials Registry (number UMIN000030786). The study protocol has been reported elsewhere^32,33^. Approximately 100,000 pregnant women participated in the JECS, and the infants will be followed up until they turn 13 years old. The recruitment was conducted between January 2011 and March 2014 at 15 Regional Centers. We excluded those with multiple participations, multiple births, miscarriages or stillbirths, congenital anomalies, missing information on maternal age at delivery, birth at <37 or ≥42 gestational weeks, unanswered questions on infant sleep quality or temperament at 1-month-old, and absence in the survey conducted when the children turned 3 years old (Fig. 1). Infants were considered 1 month of age when they were 30 or 31 days old.

### Data collection

The caregivers responded to self-administered questionnaires during the first and second or third trimesters of pregnancy and the first month and third year after birth. In addition, the infants’ medical record transcriptions at birth were collected.

#### Infant sleep quality assessment

We assessed the infants’ sleep quality at 1 month of age based on the awakening frequency and length of daytime and nighttime sleep^34^. All sleep quality data were evaluated based on the caregivers’ self-reported answers regarding the infants’ sleep. The caregivers checked the time-frames (from 00:00 to 23:30, with 30-min intervals) on the answer sheet when the infant was asleep on the day before data collection. Then, we considered the frequency of sleep interruption as “awakening” and the duration for which the infant slept as “length of sleep.” We considered ≥5 nocturnal awakenings (between 8:00 p.m. and 7:59 a.m.) as a high awakening frequency, based on the awakening frequency range (1.0–5.0 between 8:00 p.m. and 7:59 a.m.) for 2-week-old neonates^35^. We compared the lengths of daytime and nighttime sleep based on the sleep duration in the nighttime (8:00 p.m.–7:59 a.m.) and daytime (8:00 a.m.–7:59 p.m.)^36^. We defined a longer daytime than nighttime sleep period as unusual.

#### Infant temperament assessment

The behaviors recognized in autistic infants are characterized by unresponsive and irritable/overreactive types^37^. We focused on the latter type, which appeared to include more sleep problems, whereas the former type involved continuous sleep^34^. Thus, we assessed the infants’ temperaments based on their mood, crying frequency, and crying intensity^34^. The caregivers’ self-reported answers were used for assessment. The infants’ mood was identified by the question “frequency of having difficulty while holding the baby due to his/her affect and/or behavior (e.g., crying and bending backward).” The possible answers were “often,” “sometimes,” “seldom,” and “never.” We considered “often” as an indicator of a “bad mood.” The question on crying occurrence was “intensity and frequency of crying (baby),” and the answers were “quite often and long,” “sometimes and short,” or “hardly.” We considered “quite often and long” to mean “frequent crying, for long periods.” The crying intensity was identified by the question, “I have trouble calming down my crying baby.” The answers were “yes” or “no.” We considered “yes” to mean “intense crying.” All these categorizations have been defined in previous studies^23,31^.

#### ASD assessment

We identified autistic infants based on the caregivers’ answers on whether their infant was diagnosed with ASD (e.g., autism, pervasive developmental disorder, and Asperger syndrome) by the age of 3 years. This assessment was used because the study’s questionnaire was developed during the transitioning period of DSM -IV^38^ to DSM-5^2^.

#### Covariates

The covariates adjusted in the regression models were the infant’s sex, small for gestational age (<10th percentile of birth weight standards by gestational age^39^), breastfeeding status at 1 month after birth, as well as mother’s age at delivery, smoking habits, alcohol consumption, gestational age at delivery, parity, educational background, household income, and postpartum depressive symptoms at 1 month after delivery (Edinburgh Postnatal Depression Scale score^40–42^ ≥ 9). We selected the covariates adjusted with the previous study related to the association between sleep problems and temperament of 1-month-old infants^29^.

### Statistics and reproducibility

We analyzed all the data using descriptive statistics and then conducted log-binominal regression analyses to estimate the risk ratios (RRs) of ASD concerning sleep quality (≥5 nighttime awakenings and longer daytime than nighttime sleep) and temperament (bad mood, frequent crying for a long period, and intense crying). For regression analyses, we constructed two models. Model 1 was adjusted for infants’ variables, such as infant sex, small for gestational age, and breastfeeding status at 1 month after birth. Model 2 was adjusted for maternal variables, such as maternal age at delivery, smoking habits, alcohol consumption, gestational age at delivery, parity, educational background, household income, and postpartum depressive symptoms. We also conducted an analysis stratifying the participants by sex. All analyses were conducted using STATA version 16.1 (StataCorp LLC, College Station, TX, USA). The dataset used for this study was the jecs-ta-20190930 dataset released in September 2019.

### Ethical approval

The JECS protocol was reviewed and approved by the Ministry of the Environment’s Institutional Review Board on Epidemiological Studies (No. 100910001) and the Ethics Committees of all participating institutions: the National Institute for Environmental Studies that leads the JECS, the National Center for Child Health and Development, Hokkaido University, Sapporo Medical University, Asahikawa Medical College, Japanese Red Cross Hokkaido College of Nursing, Tohoku University, Fukushima Medical University, Chiba University, Yokohama City University, University of Yamanashi, Shinshu University, University of Toyama, Nagoya City University, Kyoto University, Doshisha University, Osaka University, Osaka Medical Center and Research Institute for Maternal and Child Health, Hyogo College of Medicine, Tottori University, Kochi University, University of Occupational and Environmental Health, Kyushu University, Kumamoto University, University of Miyazaki, and University of Ryukyu. The study was conducted following the Declaration of Helsinki and Japan’s Ethical Guidelines for Epidemiological Research issued by the Japanese Ministry of Education, Culture, Sports, Science, and Technology and the Ministry of Health, Labor, and Welfare. Written informed consent was obtained from all participants.

### Reporting summary

Further information on research design is available in the Nature Portfolio Reporting Summary linked to this article.

## Results

Out of the 103,060 registered pregnancies, we analyzed data from 69,751 eligible maternal and infant dyads (Fig. 1). In total, 320 (0.5%) infants were diagnosed with ASD, of whom 247 (0.4%) were male and 73 (0.1%) were female. As presented in Tables 1, 64.2% of mothers were ≥30 years old. The highest percentage of mothers had 12–16 years of schooling (64.7%), and 67.3% had household incomes between 2 and 6 million yen. More than half of mothers had already had children. Those who had ever smoked were 49.4% and had ever consumed alcohol were 65.4%. Those who had postnatal depressive symptoms were 13.7%. Regarding infants, as indicated in Table 2, around half were male. Those who were categorized in a small for gestational age were 7.2% and a majority of infants were born at 38–40 weeks gestation. Of all 4429 infants (6.3%) had ≥5 nighttime awakenings, and 13,141 (18.8%) slept longer during the day than at night. As for temperament, 4381 (6.3%) experienced bad moods; 12,109 (17.4%), frequent crying for long periods; and 13,826 (19.8%), intense crying.

**Table 1:** Baseline characteristics of mothers according to infant sleep quality and temperament categories in the Japan Environment and Children’s Study (2011–2014).

	Total		Five or more awakenings during the night		Longer daytime sleep than nighttime		Bad mood		Frequent long crying		Intense crying	
	*n*^a^	(%)	*n*^a^	(%)	*n*^a^	(%)	*n*^a^	(%)	*n*^a^	(%)	*n*^a^	(%)
Total No. of infants in the sample	69,751		4429	6.3	13,141	18.8	4381	6.3	12,109	17.4	13,826	19.8
Age at delivery (years)												
<25	5,859	8.4	282	6.4	1,250	9.5	353	8.1	983	8.1	1185	8.6
25–29	19,097	27.4	1163	26.3	3591	27.3	1237	28.2	3191	26.4	3815	27.6
30–34	25,248	36.2	1655	37.4	4649	35.4	1548	35.3	4289	35.4	4859	35.1
≥35	19,547	28.0	1329	30.0	3651	27.8	1243	28.4	3646	30.1	3967	28.7
Educational background (years)												
<10	2555	3.7	147	3.3	505	3.9	134	3.1	413	3.4	419	3.1
10–12	20,728	30.0	1218	27.7	3977	30.5	1277	29.3	3485	29.0	3752	27.3
13–16	44,761	64.7	2960	67.3	8382	64.3	2865	65.8	7913	65.9	9289	67.7
≥17	1100	1.6	71	1.6	180	1.4	77	1.8	197	1.6	263	1.9
Household income (million Japanese yen/year)												
<2	3208	4.9	214	5.1	648	5.3	198	4.8	558	5.0	574	4.5
2 to <4	21,903	33.7	1326	31.8	4226	34.6	1366	33.3	3742	33.3	4248	32.9
4 to <6	21,798	33.6	1478	35.4	4142	33.9	1415	34.5	3761	33.5	4307	33.4
6 to <8	10,742	16.5	646	15.5	1892	15.5	675	16.4	1919	17.1	2223	17.2
8 to <10	4477	6.9	292	7.0	775	6.3	299	7.3	790	7.0	980	7.6
≥10	2,848	4.4	214	5.1	548	4.5	153	3.7	468	4.2	578	4.5
Parity												
0	31,408	45.2	1,658	37.6	7003	53.5	3463	79.5	7759	64.3	9836	71.4
≥1	38,098	54.8	2748	62.4	6087	46.5	895	20.5	4300	35.7	3936	28.6
Gestational age at delivery (week)												
37	6691	9.6	480	10.8	1495	11.4	320	7.3	945	7.8	1087	7.9
38	16,012	23.0	1180	26.6	3254	24.8	844	19.3	2431	20.1	2747	19.9
39	20,657	29.6	1366	30.8	3770	28.7	1231	28.1	3600	29.7	3999	28.9
40	19,574	28.1	1086	24.5	3467	26.4	1394	31.8	3715	30.7	4268	30.9
41	6817	9.8	317	7.2	1155	8.8	592	13.5	1418	11.7	1725	12.5
Smoking habits												
Never smoked	42,218	60.7	2674	60.5	7834	59.8	2795	63.9	7475	61.8	8605	62.4
Ex-smokers who quit before pregnancy	16,342	23.5	1102	24.9	3043	23.2	893	20.4	2668	22.1	2955	21.4
Smokers during early pregnancy	11,035	15.9	644	14.6	2233	17.0	686	15.7	1947	16.1	2240	16.2
Alcohol consumption												
Never drank	24,166	34.7	1474	33.3	4652	35.4	1475	33.7	4177	34.5	4529	32.8
Ex-drinkers who quit before pregnancy	12,643	18.2	824	18.6	2279	17.4	705	16.1	2008	16.6	2282	16.5
Drinkers during early pregnancy	32,854	47.2	2125	48.0	6200	47.2	2197	50.2	5910	48.9	6999	50.7
Postpartum depressive symptoms one month after delivery were assessed by the Edinburgh Postnatal Depression Scale												
No (score <8)	59,431	86.3	3806	86.8	10,942	84.4	2985	69.2	9007	75.4	10,288	75.5
Depressive (score ≥ 9)	9424	13.7	579	13.2	2026	15.6	1331	30.8	2938	24.6	3347	24.6

^a^Subgroup totals do not equal the overall number because of missing data.

**Table 2:** Baseline characteristics of infants according to infant sleep quality and temperament categories in the Japan Environment and Children’s Study (2011–2014).

	Total		Five or more awakenings during the night		Longer daytime sleep than nighttime		Bad mood		Frequent long crying		Intense crying	
	*n*^a^	(%)	*n*^a^	(%)	*n*^a^	(%)	*n*^a^	(%)	*n*^a^	(%)	*n*^a^	(%)
Total No. of infants in the sample	69,751		4429	6.3	13,141	18.8	4381	6.3	12,109	17.4	13,826	19.8
Infant sex												
Male	35,514	50.9	2402	54.2	6529	49.7	2452	56.0	6642	54.9	7117	51.5
Female	34,237	49.1	2027	45.8	6612	50.3	1929	44.0	5467	45.2	6709	48.5
Small for gestational age												
No	64,519	92.8	4075	92.5	12,099	92.4	4044	92.8	11,091	92.0	12,670	92.0
Yes	4987	7.2	331	7.5	991	7.6	314	7.2	968	8.0	1102	8.0
Feeding status at one-month-old after birth												
Exclusive breastfeeding	37,045	54.4	2909	66.8	6501	50.6	1888	44.1	5142	43.6	6171	45.7
Partial breastfeeding	28,843	42.3	1393	32.0	5888	45.9	2186	51.1	6106	51.8	6663	49.3
Formula feeding	2231	3.3	56	1.3	450	3.5	203	4.8	546	4.6	670	5.0

^a^Subgroup totals do not equal the overall number because of missing data.

### Risk ratio of ASD according to infant sleep quality

As presented in Table 3, infants who slept longer in the daytime at 1 month old were at a high risk of ASD by 3 years old after adjusting for infant and maternal factors (RR: 1.33, 95% confidence interval [CI]: 1.01–1.75). Those with ≥5 nighttime awakenings did not significantly differ in ASD risk.

**Table 3:** Association of infant sleep quality and temperament at 1 month of age with a diagnosis of autism spectrum disorder by 3 years of age.

	Total No. of participants	No. of autism	%	Crude			Model 1^a^			Model 2^b^		
				RR.	95% CI		RR	95% CI		RR	95% CI	
Sleep												
Five or more awakenings during the night												
No	63,855	284	0.4	Reference			Reference			Reference		
Yes	4429	26	0.6	1.32	0.88	1.97	1.43	0.96	2.14	1.36	0.89	2.09
Longer daytime sleep than nighttime												
No	54,956	232	0.4	Reference			Reference			Reference		
Yes	13,141	75	0.6	1.35	1.04	1.75	1.38	1.06	1.79	1.33	1.01	1.75
Temperament												
Bad mood												
No	65,249	283	0.4	Reference			Reference			Reference		
Yes	4381	37	0.8	1.95	1.38	2.74	1.79	1.27	2.53	1.35	0.92	1.97
Frequent long crying												
No	57,387	242	0.4	Reference			Reference			Reference		
Yes	12,109	78	0.6	1.53	1.18	1.97	1.34	1.03	1.74	1.21	0.92	1.60
Intense crying												
No	55,711	228	0.4	Reference			Reference					
Yes	13,826	92	0.7	1.63	1.28	2.07	1.51	1.18	1.93	1.31	1.00	1.72

*CI* confidence interval, *RR* risk ratio.

^a^Adjusted for infant sex, small for gestational age, and feeding status one month after birth.

^b^Additionally adjusted for maternal age at delivery, smoking habits, alcohol consumption, gestational age at delivery, parity, educational background, household income, and postpartum depressive symptoms at 1 month after delivery.

### Risk ratio of ASD according to infant temperament

As presented in Table 3, after adjusting for infant factors, it was revealed that infants who experienced bad moods (RR: 1.79, 95% CI: 1.27–2.53) and those who experienced frequent long crying (RR: 1.34, 95% CI: 1.03–1.74) had a higher risk of ASD by 3 years old. Those who experienced intense crying had a higher risk of ASD after adjusting for infant and mother factors (RR: 1.31, 95% CI: 1.00–1.72).

### Sex-stratified RR of ASD

Table 4 presents the sex-stratified RR of ASD. The risk of association between a bad mood at 1 month of age and later ASD significantly varied between sex (*P* < 0.001). Female infants with bad moods had a higher risk of later ASD (RR: 3.59, 95% CI: 1.91–6.75) after adjusting for infant and mother factors. However, there was no significant increase in relative risk for ASD diagnosis associated with the mood in male infants (RR: 0.90, 95% CI: 0.55–1.47). Male infants who experienced intense crying were at increased risk of ASD after adjusting for infant factors (RR: 1.45, 95% CI: 1.09–1.92). Female infants who experienced intense crying were at a higher risk of ASD after adjusting for infant factors (RR: 1.75, 95% CI: 1.05–2.91).

**Table 4:** Sex-stratified association of infant sleep quality and temperament at 1 month of age with a diagnosis of autism spectrum disorder by 3 years of age.

	Male infants (*n* = 35,514)									Female infants (*n* = 34,237)									The *P* value for effect modification
	Total No. of participants	No. of autism	%	Model 1^a^			Model 2^b^			Total No. of participants	No. of outcome		Model 1^a^			Model 2^b^			
				RR.	95% CI		RR	95% CI				%	RR.	95% CI		RR	95% CI		
Sleep																			
Five or more awakenings during the night																			
No	32,364	221	0.7	Reference			Reference			31,491	63	0.2	Reference			Reference			
Yes	2402	20	0.8	1.35	0.85	2.13	1.24	0.76	2.04	2027	6	0.3	1.79	0.77	4.15	1.92	0.82	4.48	0.46
Longer daytime sleep than nighttime																			
No	28,141	182	0.7	Reference	Reference	26,815	50	0.2											
Yes	6529	57	0.9	1.33	0.99	1.80	1.26	0.92	1.73	6612	18	0.3	1.54	0.89	2.66	1.57	0.90	2.73	0.38
Temperament																			
Bad mood																			
No	32,998	225	0.7	Reference			Reference			32,251	58	0.2	Reference			Reference			
Yes	2452	22.0	0.9	1.28	0.83	1.99	0.90	0.55	1.47	1929	15	0.8	4.27	2.41	7.56	3.59	1.91	6.75	<0.001*
Frequent long crying																			
No	28,747	187	0.7	Reference			Reference			28,640	55	0.2	Reference			Reference			
Yes	6642	60	0.9	1.32	0.98	1.77	1.20	0.88	1.64	5467	18	0.3	1.44	0.82	2.52	1.24	0.68	2.26	0.74
Intense crying																			
No	28,287	179	0.6	Reference			Reference			27,424	49	0.2	Reference			Reference			
Yes	7117	68	1.0	1.45	1.09	1.92	1.24	0.91	1.69	6709	24	0.4	1.75	1.05	2.91	1.58	0.90	2.76	0.37

*CI* confidence interval, *RR* risk ratio. *<0.01.

^a^Adjusted for small for gestational age and feeding status 1 month after birth.

^b^Additionally adjusted for maternal age at delivery, smoking habits, alcohol consumption, gestational age at delivery, parity, educational background, household income, and postpartum depressive symptoms at 1 month after delivery.

## Discussion

To the best of our knowledge, this is the first study to demonstrate an association between sleep quality and temperament at 1 month of age and later ASD. Infants who slept for longer during the day at 1 month old showed a higher risk of developing ASD by age three. Bad moods and frequent, persistent, or intense crying at 1 month of age were also associated with a higher risk of ASD by 3 years old, after adjusting for infant-related factors. A sex-stratified analysis found the sex of the infant not affecting sleep. However, frequent bad moods were an ASD risk factor in female, but not male, infants. We also identified a slightly higher tendency to intense crying in females than males. These associations may be early prognostic indicators of later ASD.

Our study demonstrated that a longer daytime sleep pattern at 1 month of age was associated with a later ASD diagnosis. A similar result was obtained in a previous retrospective study among 1–6-year-old children^34^. Also, our study demonstrated that the association of autism with the sleep of infants is evident at an earlier age than that reported in a previous study. This association supports the suggestion by Mike et al. that fetal development of circadian chronobiology may be immature or disrupted in infants with sleep problems^34^. This is thought to result from low maternal melatonin secretion, which has been shown to increase the risk that the fetus will later develop ASD^43^. Problems with clock-controlled genes have also been reported to disturb sleep cycle rhythms in autistic children^44^. Some of these genes (e.g., PER1, NPAS2) were found to be associated with ASD development and sleep disturbances in a study of autistic children and parents^45^. Sleep problems may also lead to the development of an irritable or overreactive temperament in infants^29^, which, in turn, increases the risk of later ASD^34^. Our findings supported this relationship between difficult temperament and later ASD. However, Nguyen et al. found no association between infant sleep quality at 12 months, either at night or during the day, with ASD-type behaviors at 24 months, as identified using the modified checklist for autism in toddlers (M-CHAT)^22^. This differs from our findings. This discrepancy may be because, as stated in the Discussion section of the Nguyen et al. study, M-CHAT scores may not provide sufficient accuracy for a precise determination of ASD risk. Further research is needed to confirm this association.

We found that female infants who exhibited frequent bad moods had a higher risk of later ASD. There are known to be sex-specific differences in infant temperament, including higher reactivity to perceived environmental threats in female than male infants^31,46,47^. Such sex-specific dispositions might have affected some characteristics of subsequent ASD. Further studies are needed to elucidate sex differences involving temperament and ASD, as they could be important indicators of ASD in female infants.

In our analysis, a difficult temperament at 1 month of age, as evidenced by bad moods and frequent crying was associated with an increased risk of ASD by the age of three. This has been reported in previous studies;^48,49^ however, these have not studied infants younger than 6 months^50^. Thus, our findings add to the literature by evidencing risk factors for ASD in 1-month-old infants^51^. An interesting finding of our study was that there was no significant relationship between infant temperament and later ASD development when the mothers’ covariates were included in the assessed model. As indicated in the descriptive analyses of the participants, mothers who perceived their infant to have a poor temperament were more likely to be primiparous, had more depressive symptoms, and were less likely to be breastfeeding than those who did not. Such factors may have moderated the association between temperament and ASD. For example, primiparous mothers may react more to the duration and intensity of infant crying than multiparous mothers^52^. Also, maternal factors related to the risk for later development of autism will differ between mothers. For example, previous studies suggested both maternal smoking^53^ and maternal drinking during^54^ pregnancy were linked to the later development of autism in the child. However, according to meta-analyses, current evidence does not support any independent association between alcohol consumption and ASD^54^.

This study, using a large sample size, addressed the difficulty of ASD diagnosis by demonstrating that infant sleep quality and temperament at only 1 month of age may be associated with ASD development. Such behaviors can be observed even by caregivers and can thus enable early intervention. However, this study had some limitations. First, the assessment of sleep was based on caregivers’ reports. Since these are external observations of sleep, their accuracy is not guaranteed. Reports of infant sleep quality might be overestimated if there is no behavioral evidence of poor sleep; whereas, the rate of night awakenings may be underestimated due to brief awakenings in which the infant does not cry and wake their caregiver^55^. Therefore, the caregivers’ responses may have influenced our study findings. Second, ASD outcome was identified according to caregivers’ self-report on the infants’ ASD diagnosis. Therefore, infants who might be subsequently diagnosed with ASD were not considered, and the actual number might be underestimated as the rate in this study was lower than in another study in Japan^56^. Third, because the data were obtained from a large-scale study, minor differences may have exhibited significance. Fourth, this study may not have covered all infant temperaments and could be improved in future research.

In conclusion, this study demonstrated that infant sleep patterns and temperament are associated with the risk that the infant will develop ASD before the age of three. Specifically, infants prone to longer daytime sleeping; more frequent bad moods; and intense, frequent, or prolonged crying showed a higher incidence of ASD at 3 years old. In particular, the association between a bad mood and ASD was characteristic of female infants. These findings may serve as important indicators for identifying infants requiring intervention to reduce the risk of future ASD.

## Supplementary information

Reporting Summary

## Data Availability

Data are unsuitable for public deposition due to ethical restrictions and the legal framework of Japan. It is prohibited by the Act on the Protection of Personal Information (Act No. 57 of May 30 2003, amendment on 9 September 2015) to deposit the data containing personal information publicly. Ethical Guidelines for Medical and Health Research Involving Human Subjects enforced by the Japan Ministry of Education, Culture, Sports, Science and Technology and the Ministry of Health, Labor and Welfare also restrict the open sharing of epidemiologic data. All inquiries about access to data should be sent to: jecs-en@nies.go.jp. The person responsible for handling inquiries sent to this e-mail address is Dr. Shoji F. Nakayama, JECS Programme Office, National Institute for Environmental Studies.
