# Supplementary material for: Sleep quality and temperament in association with autism spectrum disorder among infants in Japan
Source: Commun Med (Lond). 2023 Jun 16;3:82. doi: 10.1038/s43856-023-00314-9 (PMC10275966; doi:10.1038/s43856-023-00314-9)
Supplement: Supplementary file 1 — Reporting Summary [file 43856_2023_314_MOESM1_ESM.pdf]

nature portfolio

Corresponding author(s): Seiichi Morokuma

Last updated by author(s): Feb 3, 2023

Reporting Summary

Nature Portfolio wishes to improve the reproducibility of the work that we publish. This form provides structure for consistency and transparency in reporting. For further information on Nature Portfolio policies, see our Editorial Policies and the Editorial Policy Checklist.

Statistics

For all statistical analyses, confirm that the following items are present in the figure legend, table legend, main text, or Methods section.

n/a	Confirmed	
☐	☒	The exact sample size (n) for each experimental group/condition, given as a discrete number and unit of measurement
☐	☒	A statement on whether measurements were taken from distinct samples or whether the same sample was measured repeatedly
☐	☒	The statistical test(s) used AND whether they are one- or two-sided<br>*Only common tests should be described solely by name; describe more complex techniques in the Methods section.*
☐	☒	A description of all covariates tested
☐	☒	A description of any assumptions or corrections, such as tests of normality and adjustment for multiple comparisons
☐	☒	A full description of the statistical parameters including central tendency (e.g. means) or other basic estimates (e.g. regression coefficient) AND variation (e.g. standard deviation) or associated estimates of uncertainty (e.g. confidence intervals)
☒	☐	For null hypothesis testing, the test statistic (e.g. F, t, r) with confidence intervals, effect sizes, degrees of freedom and P value noted<br>*Give P values as exact values whenever suitable.*
☒	☐	For Bayesian analysis, information on the choice of priors and Markov chain Monte Carlo settings
☒	☐	For hierarchical and complex designs, identification of the appropriate level for tests and full reporting of outcomes
☒	☐	Estimates of effect sizes (e.g. Cohen's d, Pearson's r), indicating how they were calculated

Our web collection on statistics for biologists contains articles on many of the points above.

Software and code

Policy information about availability of computer code

Data collection	We do not have commercial, open source and custom code for data collection.
Data analysis	All analyses were conducted using STATA version 16.1 (StataCorp LLC, College Station, TX, USA).

For manuscripts utilizing custom algorithms or software that are central to the research but not yet described in published literature, software must be made available to editors and reviewers. We strongly encourage code deposition in a community repository (e.g. GitHub). See the Nature Portfolio guidelines for submitting code & software for further information.

Data

Policy information about availability of data

All manuscripts must include a data availability statement. This statement should provide the following information, where applicable:

- Accession codes, unique identifiers, or web links for publicly available datasets
- A description of any restrictions on data availability
- For clinical datasets or third party data, please ensure that the statement adheres to our policy

Data are unsuitable for public deposition due to ethical restrictions and the legal framework of Japan. It is prohibited by the Act on the Protection of Personal Information (Act No. 57 of May 30 2003, amendment on 9 September 2015) to deposit the data containing personal information publicly. Ethical Guidelines for Medical and Health Research Involving Human Subjects enforced by the Japan Ministry of Education, Culture, Sports, Science and Technology and the Ministry of

Health, Labor and Welfare also restrict the open sharing of epidemiologic data. All inquiries about access to data should be sent to: jecs-en@nies.go.jp. The person responsible for handling inquiries sent to this e-mail address is Dr. Shoji F. Nakayama, JECS Programme Office, National Institute for Environmental Studies.

Human research participants

Policy information about studies involving human research participants and Sex and Gender in Research.

Reporting on sex and gender	We applied parents' reported infant's male and female sex and used the term "sex" for indicating biologial attribution of the subjects. We reported sex-based analyses.
Population characteristics	We included covariate-relevant population characteristics of the human research participants such as small for gestational age, breastfeeding status of child, maternal age at delivery, maternal smoking habits, maternal alcohol consumption, gestational age at birth, parity, educational background, household income, and postpartum depressive symptoms.
Recruitment	The recruitment was conducted between January 2011 and March 2014 at 15 Regional Centers of the Japan Environment and Children's Study. This was a large cohort study which allow to minimize the participant bias.
Ethics oversight	The JECS protocol was reviewed and approved by the Ministry of the Environment's Institutional Review Board on Epidemiological Studies (No. 100910001) and the Ethics Committees of all participating institutions.

Note that full information on the approval of the study protocol must also be provided in the manuscript.

Field-specific reporting

Please select the one below that is the best fit for your research. If you are not sure, read the appropriate sections before making your selection.

☐ Life sciences ☒ Behavioural & social sciences ☐ Ecological, evolutionary & environmental sciences

For a reference copy of the document with all sections, see nature.com/documents/nr-reporting-summary-flat.pdf

Behavioural & social sciences study design

All studies must disclose on these points even when the disclosure is negative.

Study description	Quantitative longitudinal study.
Research sample	Samples are mothers and infants from a large-cohort study, the Japan Environment and Children's Study (JECS). Approximately 100,000 pregnant women participated in the JECS, and the infants will be followed up until they turn 13 years old. The total enrolled pregnant women of this study per year was around 3% of total Japanese newborns per year. The recruitment was conducted between January 2011 and March 2014 at 15 Regional Centers.
Sampling strategy	We contacted with as many expecting mothers who reside in study areas as possible. The recruitment rate is targeted to be more than 50% of all eligible mothers. Either or both of the following two recruitment protocols are applied: 1) recruitment at the time of first prenatal examination at cooperating health care providers, 2) recruitment at local government offices issuing pregnancy journals.
Data collection	Questionnaires are administered to enrolled mothers and their partners at first trimester and second/third trimester in pregnancy. Medical histories of past and present pregnancies are recorded.
Timing	The participating children are followed up until they reach the age of 13 years.
Data exclusions	We excluded those with multiple participations, multiple births, miscarriages or stillbirths, congenital anomalies, missing information on maternal age at delivery, births at <37 or ≥42 gestational weeks, unanswered questions on infant sleep quality or temperament at 1 month old, and abstinence in the survey conducted when the children turned 3 years old (Figure 1). Infants were considered 1 month old when they were 30 or 31 days old.
Non-participation	Excluded are those who do not consent to the study protocol and cannot be accessed during the pregnancy period.
Randomization	The recruitment is not completely random.

Reporting for specific materials, systems and methods

We require information from authors about some types of materials, experimental systems and methods used in many studies. Here, indicate whether each material, system or method listed is relevant to your study. If you are not sure if a list item applies to your research, read the appropriate section before selecting a response.

Materials & experimental systems

n/a	Involved in the study
☒	☐ Antibodies
☒	☐ Eukaryotic cell lines
☒	☐ Palaeontology and archaeology
☒	☐ Animals and other organisms
☒	☐ Clinical data
☒	☐ Dual use research of concern

Methods

n/a	Involved in the study
☒	☐ ChIP-seq
☒	☐ Flow cytometry
☒	☐ MRI-based neuroimaging
